# Validation of an equation for energy expenditure that does not require the respiratory quotient

**DOI:** 10.1371/journal.pone.0211585

**Published:** 2019-02-01

**Authors:** Karl J. Kaiyala, Brent E. Wisse, John R. B. Lighton

**Affiliations:** 1 Department of Oral Health Sciences, School of Dentistry, University of Washington, Seattle, Washington United States of America; 2 Department of Medicine, University of Washington, Seattle Washington, United States of America; 3 Sable Systems International Inc., North Las Vegas, Nevada, United States of America; St. Joseph's Hospital and Medical Center, UNITED STATES

## Abstract

**Background:**

Energy expenditure (EE) calculated from respirometric indirect calorimetry is most accurate when based on oxygen consumption (VO_2_), carbon dioxide production (VCO_2_) and estimated protein metabolism (PM). EE has a substantial dependence of ~7% on the respiratory quotient (RQ, VCO_2_/VO_2_) and a lesser dependence on PM, yet many studies have instead estimated EE from VO_2_ only while PM has often been ignored, thus reducing accuracy. In 1949 Weir proposed a method to accurately calculate EE without using RQ, which also adjusts for estimated PM based on dietary composition. This RQ^-^ method utilizes the calorimeter airflow rate (FR), the change in fractional O_2_ concentration (ΔFO_2_) and the dietary protein fraction. The RQ^-^ method has not previously been empirically validated against the standard RQ^+^ method using both VO_2_ and RQ. Our aim was to do that.

**Methods:**

VO_2_ and VCO_2_ were measured repeatedly in 8 mice fed a high protein diet (HPD) during exposure to different temperatures (n = 168 measurements of 24h gas exchange). The HPD-adjusted RQ^+^ equation was: EE [kcal/time] = VO_2_ [L/time]×(3.853+1.081RQ) while the corresponding RQ^-^ equation was: EE = 4.934×FR×ΔFO_2_. Agreement was analyzed using the ratios of the RQ^-^ to RQ^+^ methods along with regression and Bland-Altman agreement analyses. We also evaluated the standard equation using the dietary food quotient (FQ) of 0.91 as a proxy for RQ (FQ^+^ method).

**Results:**

Ratio analysis revealed that the mean error of the RQ^-^ method was only 0.11 ± 0.042% while the maximum error was only 0.21%. Error using the FQ^+^ method was 4 -and 10-fold greater, respectively. Bland-Altman analysis demonstrated that the RQ^-^ method very slightly overestimates EE as RQ decreases. Theoretically, this error can be eliminated completely by imposing an incurrent fractional oxygen concentration at a value only slightly greater than the atmospheric level.

**Conclusions:**

The Weir ‘RQ-free’ method for calculating EE is a highly valid alternative to the ‘gold standard’ method that requires RQ. The RQ^-^ approach permits reduced cost and complexity in studies focused on EE and provides a way to rescue EE measurement in studies compromised by faulty CO_2_ measurements. Practitioners of respirometry should consider adjusting EE calculations for estimated protein metabolism based on dietary composition.

## Introduction

Animal life is powered by catalytic combustion, the intricate “Fire of Life” [1] by which oxygen necessarily participates in the thermochemical transformation of food energy into biological work and heat. In accord with Hess’s law of constant heat sums (a restatement of the first law of thermodynamics) [2], the quantity of heat released by the low temperature biological “fire” is the same as when an equivalent amount food is suddenly combusted in a bomb calorimeter. Accordingly, in the 242 years since Lavoisier first proposed an obligatory metabolic role for “oxygene” [2], technological and conceptual advances have steadily improved the ability to quantify energy expenditure (EE) using mathematical transformations of oxygen consumption (VO_2_), carbon dioxide production (VCO_2_), or both to achieve the greatest accuracy [1–5].

The ratio of VCO_2_ to VO_2_, termed the respiratory quotient (RQ), normally ranges between ~0.7 and ~1.0 depending largely on the proportions of carbohydrate, fat and protein being combusted. EE is commonly calculated by multiplying VO_2_ times a linear transform of the form (A+B×RQ), where A and B are coefficients whose ‘most accurate’ values have a modest inverse dependence on the rate of protein metabolism as estimated from nitrogen excretion or diet composition [6, 7]. By contrast, the per liter energy equivalent of VO_2_ depends more importantly on RQ: when RQ equals 0.7, each liter of VO_2_ represents ~4.7 kcal, but when RQ equals 1.0 the value is ~5 kcal. It is, therefore, widely understood that the most accurate estimation of EE from respiratory gas exchange requires measurements of both VO_2_ and VCO_2_.

One of the most widely employed equations for calculating EE from respirometric data was introduced in 1949 by Weir in a paper focused principally on adjusting the EE calculation for protein metabolism [6]:

$$EE=\dot{V}O_{2}\frac{\left(3.941+1.106(RQ)\right)}{\left(1+0.082P\right)}$$ [1]

where *P* is the proportion of total EE accounted for by protein metabolism [6, 7]. EE is in units of kcal/h when VO_2_ is in L/h, and so forth. VO_2_ is corrected to standard temperature and pressure, dry (STPD). We denote Eq 1 the RQ^+^ method.

In the same paper [6] that introduced Eq 1, Weir also proposed a second and theoretically accurate equation for EE that relies only on the airflow rate and change in fractional oxygen concentrations; if *P* is 0.1, and the excurrent flow rate is measured, the equation is simply:

$$EE=5\times {\dot{V}}_{e}\times \Delta FO_{2}$$ [2]

where V_e_ is the excurrent flow rate corrected to STPD, and ΔFO_2_ is the incurrent minus excurrent fractional oxygen difference (F_i_O_2_ minus F_e_O_2_). Of critical importance to the validity of Eq 2 (and for the calculation of VO_2_ and RQ in Eq 1), the fractional gas concentrations must be either scrubbed of water vapor or mathematically corrected for its presence using Dalton’s law of partial pressures [3–5] to avoid dilution by this gas, which otherwise would result in marked overestimation of VO_2_, a lesser overestimation of VCO_2,_ and a consequent dramatic underestimation of RQ [3, 4] (discussed below).

It is important to emphasize that the constant term in Eq 2 depends on the fraction of total EE accounted for by protein fraction in Eq 1, as well as on the particular EE equation employed to transform gas exchange into EE (e.g., an updated equation has been proposed by Hall and colleagues [7]). Therefore, we denote Eq 2 as being representative of the RQ^-^ method.

The RQ^-^ method is all but unknown, likely overshadowed by Weir’s canonical EE equation [6] and perhaps impeded also by the somewhat arcane theoretical explanations put forward by Weir and others [3, 6, 8, 9]. Perhaps the most important reason, however, is that (to our knowledge) the RQ^-^ method has never been validated in a rigorous empirical test.

If the RQ^-^ method in fact agrees very well with the standard RQ^+^ method, its adoption could confer a number of important advantages (discussed below). Accordingly, our major aim was to validate the RQ^-^ method. We also present a new and hopefully more rigorous and transparent explanation for why the method should work, and demonstrate that, theoretically, the agreement between the RQ^-^ and RQ^+^ methods can be made perfect by imposing an incurrent oxygen fraction that is only slightly greater than the normal atmospheric value. Finally, our analysis suggests that protein metabolism should get wider consideration in the application of respirometry.

## Materials and methods

### Subjects, diet and institutional approval

Male C57Bl/6J mice (N = 8, age ~8 weeks; Jackson Laboratories (Bar Harbor, ME)) were housed in a constant temperature walk-in room housed within the AAALAC-accredited animal care facility at the University of Nevada at Las Vegas. Mice were individually housed within live-in, unsealed, pull-mode metabolic measurement cages (see [3–5] for discussions of pull- vs. push-mode respirometry). The mice were fed the Labdiet 5001 (LabDiets, St Louis, MO), a high protein diet (HPD; 28.5% of kcals; fat = 13.4% of kcals) having a food quotient (FQ) of 0.906 (calculated using the ‘Indirect Calorimetry Equations’ supplement in [7]). Mice were supplied with water ad libitum, and provided with Bed O'Cobs 1/4” bedding material (Anderson Industrial Products, Maumee, OH).

All study procedures were approved by the Institutional Animal Care and Use Committee (IACUC) at the University of Nevada at Las Vegas. The program is fully accredited by the Association for the Assessment and Accreditation for Laboratory Animal Care International (AAALAC).

### Derivation of the RQ^-^ equation for the high protein diet

A valid expression for VO_2_, easily derived from equation 11.2 on p.126 in [3], is:

$$\dot{V}O_{2}=\frac{{\dot{V}}_{e}(F_{i}O_{2}-F_{e}O_{2})}{1-F_{i}O_{2}+F_{i}O_{2}RQ}$$ [3]

(We emphasize that the flow rate and fractional gas concentrations must be mathematically corrected for or scrubbed of water vapor).

Next, to compute EE in accordance with Eq 1, we multiply VO_2_ times the Weir RQ^+^ transform adjusted for the HPD (i.e., 3.853 + 1.081RQ), but write the result as follows:

$$EE={\dot{V}}_{e}(F_{i}O_{2}-F_{e}O_{2})\frac{\left(3.853+1.081RQ\right)}{\left(1-F_{i}O_{2}+F_{i}O_{2}RQ\right)}$$ [4]

Note that we intentionally placed the RQ^+^ transform over the denominator of Eq 3 for VO_2_ and placed that quotient (Q) to the right in Eq 4 because, as is easily confirmed, Q very nearly equals 4.934 for any value of RQ ranging from 0.7 to 1.0 (note that 4.934 is the value of the RQ^+^ transform when RQ = 1.0). A formal (and to our knowledge novel) explanation for why this pertains is as follows: Multiply the numerator of Q by 1.0 but express this as the ratio 4.934÷4.934. Next, substitute the atmosphere’s near constant fractional O_2_ of 0.20939 [10] into the denominator of Q. The result can then be written as:

$$EE={\dot{V}}_{e}(F_{i}O_{2}-F_{e}O_{2})\frac{\left(0.7809+0.2191RQ\right)}{\left(0.7906+0.2094RQ\right)}(4.934)≅{\dot{V}}_{e}(F_{i}O_{2}-F_{e}O_{2})(4.934)$$ [5]

Remarkably, the numerator and denominator of the ratio in Eq 5 are nearly identical linear functions of RQ. Therefore, the ratio remains very close to 1.0 across the entire range of RQ; specifically, it equals 1.0 when RQ equals 1.0, and equals 0.9969 when RQ equals 0.7. Accordingly, and as indicated above, Eq 5 can be simplified to exclude any dependence on RQ with almost no loss of accuracy. We employed the simplified expression of Eq 5 to compute RQ^-^ EE in the present work.

It can be shown that the RQ^-^ minus RQ^+^ EE difference in kcal/h calculated as the simplified version of Eq 5 minus Eq 1 adjusted for the HPD equals the equation:

$$\Delta EE=0.0029\dot{V}O_{2}(1-RQ)$$ [6]

Thus, for a given RQ <1.0, the EE difference is predicted to scale directly but only slightly with VO_2_, and for a given VO_2_, the difference is predicted to increase linearly, but again only slightly, as RQ decreases.

### Respirometry

Metabolic rates were measured using an 8-cage Promethion-C continuous, parallel metabolic phenotyping system (Sable Systems International (SSI), Las Vegas, NV; SSI). This system imposes minimal stress due to handling or other disruptive influences because it uses live-in cages of ~8 L STP internal volume that are transferred from the housing colony to the testing room for studies. Air was pulled from the cages at a controlled mass flow rate of 2 L/min STP. This yielded a time constant of ~4 min. The flow from each cage was sampled by a gas analysis chain consisting of a water vapor analyzer a CO_2_ analyzer, an O_2_ analyzer, a barometric pressure sensor, and a subsampling flow control system, all integrated into one gas analysis system (GA3m4: SSI) per bank of 4 cages. Gas flow for each bank was generated by a FR-4b mass flow controlled pull flow generator (SSI). The calorimeter room also incorporated a fluorescent light source controlled by a timer set to a 12:12 light:dark cycle.

The system acquired data on fractional O_2_ and CO_2_ concentrations, water vapor pressure (WVP), barometric pressure (BP), ambient temperature and light levels, flow rates, food and water dispenser masses (to 1 mg), body masses (to 1 mg via a weighed enrichment habitat), running wheel revolutions, and X, Y and Z locations together with beam-breaks. Measurements were acquired at a sample rate of 1 sample/sec for all sensors and cages simultaneously via an error-correcting control area network (CAN). The provision for exercise increased variability in EE and RQ, important goals of our study design, but the primary strategy for achieving that end was to systematically vary the temperature of the testing room from 19 to 29°C. Specifically, the 8 mice were each tested at 19°C (total of 3 d), 21°C (9 d), 23°C (3 d), 25°C (2 d), 27°C (1 d), and 29°C (4 d) for a total of N = 168 measurements. Note that the maintenance of thermal homeostasis in mice requires EE to be exquisitely sensitive to even the seemingly mild cold stress imposed by typical laboratory temperatures of ~21°C while thermoneutrality in mice is achieved at ~30–32°C [11–14].

We corrected for water vapor dilution of fractional gas concentrations using Dalton's law of partial pressures, an application of elementary chemistry based on measurement of BP and WVP in the gas stream. The equation is simply:

$$F^{DRY}gas=\frac{F^{DIL}gas\;(BP)}{\left(BP‑WVP\right)}$$ [7]

where *F*^*DRY*^*gas* and *F*^*DIL*^*gas* are the WVP-corrected and diluted fractional gas concentrations, and WVP is in the same units as BP [3–5].

The calorimeter system switched from measuring the excurrent air pulled from each cage to measuring incurrent air pulled from the cages’ environment for 30 sec every 20 min. This permitted periodic re-spanning of the O_2_ analyzers to WVP-corrected F_i_O_2_ = 0.2094, effectively eliminating O_2_ drift; this also allowed measurement of F_i_CO_2_. During data analysis these brief and infrequent interruptions–each lasting only ~15% of the cage time constant, thus minimizing their effect on the underlying data -were removed by linear interpolation, which rendered them effectively invisible.

We also contrasted the performance of the RQ^-^ equation with the standard Weir equation using the mouse diet’s FQ of 0.91 as a proxy for RQ, denoted the FQ^+^ method.

### Data reduction and statistical analysis

Data were stored in raw, unprocessed form for later analysis using analysis scripts run on ExpeData analytical software (SSI). This allowed complete and traceable control of the analytical process, the equations used, the baselining algorithms employed, and all other aspects of data transformation and final data extraction.

For the major analyses in this paper, calorimeter outcomes were averaged (mean) across each 24h circadian cycle of each day of exposure at the 6 ambient temperatures. A separate time series analysis binned the 1 Hz continuous data into 30 sec bins across a 24h study to make the data and graphical analyses more tractable to analysis.

Agreement between the RQ^-^ and RQ^+^ equations was analyzed in several ways. One simply involved analysis of the N = 168 ratios of RQ^-^:RQ^+^ EE calculations. We also employed the Tukey mean-difference method (widely known as the Bland-Altman technique [15] because this citation classic [16] is considered the ‘gold standard’ for agreement analysis), and we augmented this by regression analysis. We also present scatterplots involving the methods due to their intuitive accessibility. Owing to the study’s repeated measures design, regression analyses were performed using linear mixed model analysis [17].

All statistical and graphical analyses were performed using R (R: A Language and Environment for Statistical Computing. R Core Team. R Foundation for Statistical Computing. Vienna, Austria, 2018. url = https://www.R-project.org/).

Data are reported as means ± SEM unless reported otherwise.

## Results

### Agreement analysis

Mean ± SD EE outcomes in kcal/h for the three computational methods were essentially the same: RQ^+^ = 0.421 ± 0.0651, RQ^-^ = 0.421 ± 0.0652, FQ^+^ = 0.423 ± 0.0667. The mean ± SD ratios were: (RQ^-^/RQ^+^) = 1.0011 ± 0.00042, (FQ^+^/RQ^+^) = 1.0044 ± 0.0092. The most extreme ratios were: (RQ^-^/RQ^+^) = 1.0021, (FQ^+^/RQ^+^) = 1.0265. Accordingly, the largest relative errors compared to the ‘gold standard’ RQ^+^ method were 0.21% for the RQ^-^ method and 2.65% for the FQ^+^ method.

**Fig 1:**
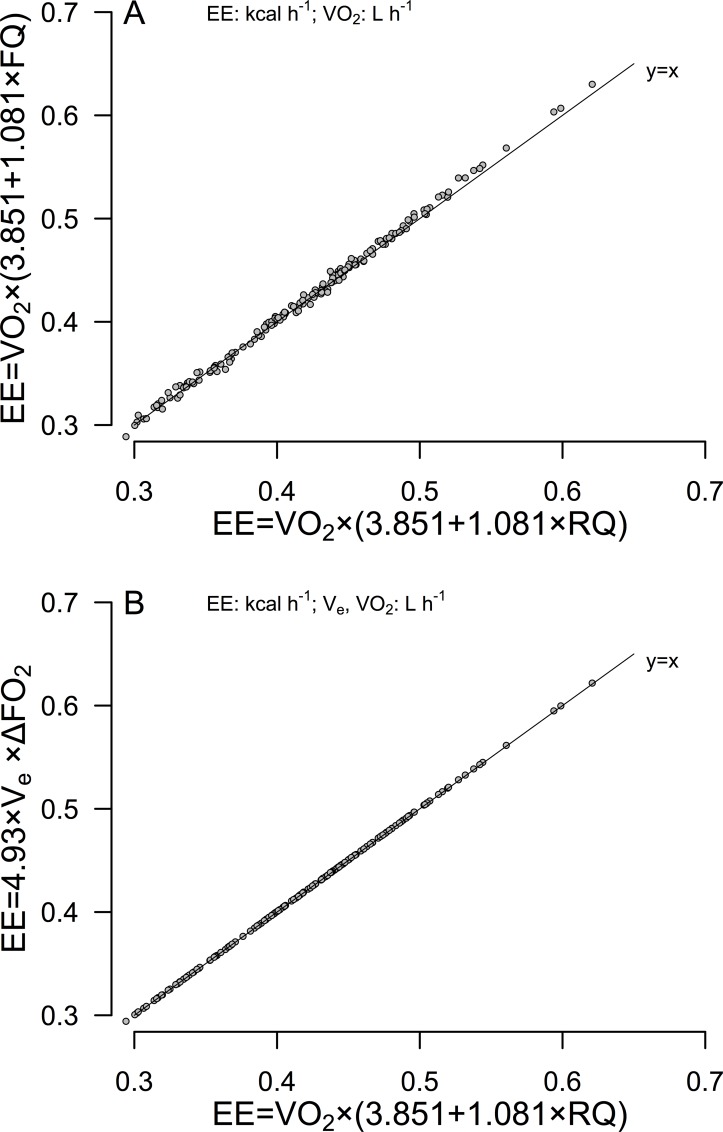
Scatterplots comparing the results of equations that do not use RQ compared to the standard Weir equation that does use RQ. (A) Substituting the FQ of the mouse chow for RQ in the standard Weir equation (y-axis) versus the standard Weir RQ^+^ equation. (B) Comparison of the Weir RQ^-^ equation to the standard Weir RQ^+^ method.

Scatterplots relating the alternative EE equations are depicted in Fig 1.

Data consist of 168 measurements of 24h EE in n = 8 mice. EE, energy expenditure; RQ, Respiratory Quotient; FQ, Food Quotient.

**Fig 2:**
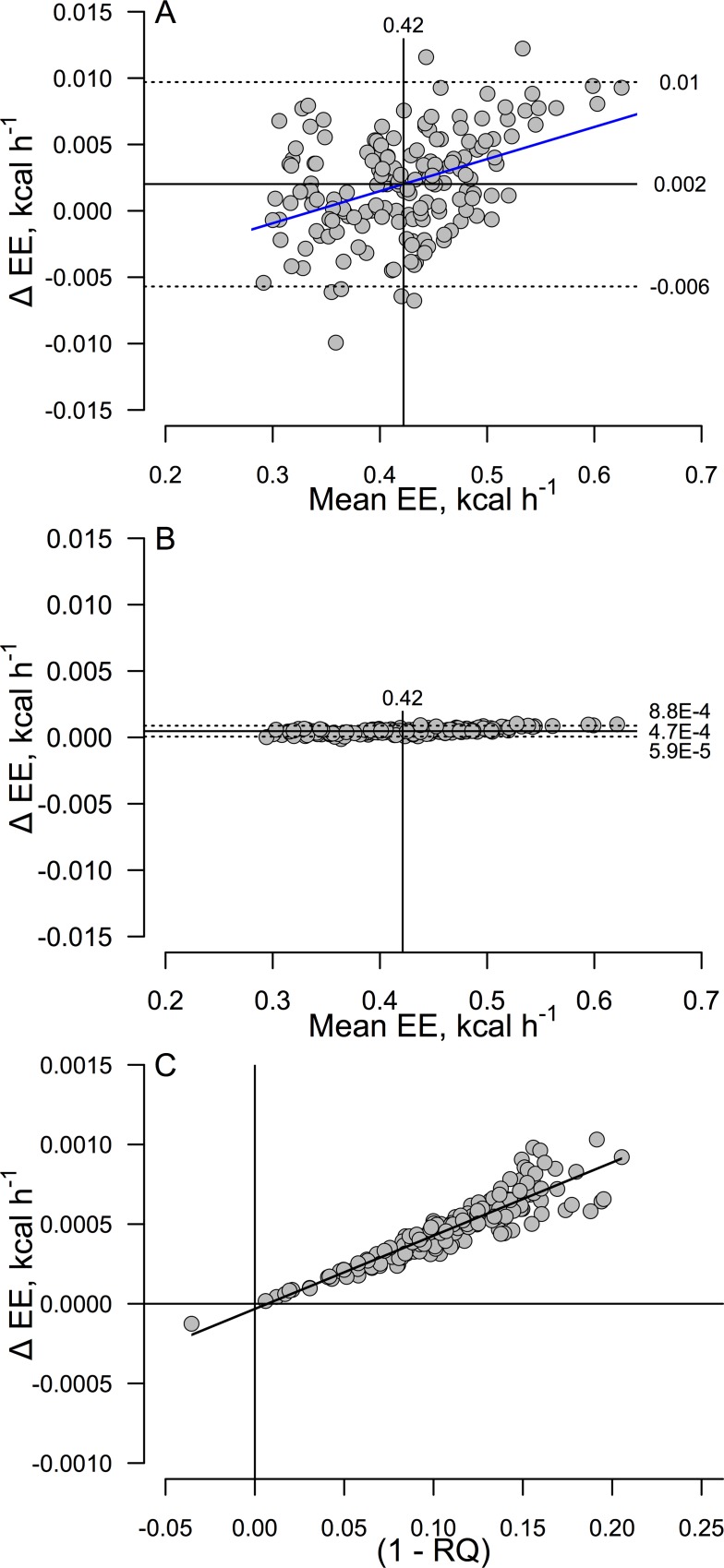
Tukey-Bland-Altman mean-difference agreement plots comparing EE computed by alternative methods, and error as a function of RQ. In panels A and B the x-axis depicts the mean of two methods being compared and the y-axis depicts the difference; the standard Weir RQ^+^ method was subtracted from the other method (refer to text or Fig 1 for equations). The plots indicate the mean bias (solid horizontal lines) ± 2 SDs (dashed lines) and mean EE (solid vertical lines). (A) Agreement between the Weir EE equation using FQ as a proxy for RQ and the standard Weir RQ^+^ EE. (B) Agreement between the Weir RQ^-^ equation and the standard Weir RQ^+^ equation. The superiority of this method over the one assuming that RQ equals FQ is striking. (C) Weir RQ^-^ minus Weir RQ^+^ EE differences as a function of 1 minus RQ. Note that the predicted mean EE difference between methods at RQ = 0.7 is just 0.0014 kcal/h, which represents 0.31% of the corresponding metabolic rate calculated from the RQ^+^ method at RQ = 0.7. Data consist of 168 measurements of 24h EE in n = 8 mice. EE, energy expenditure; RQ, Respiratory Quotient; FQ, Food Quotient; ΔEE = Weir FQ^+^ or Weir RQ^-^ minus standard the Weir RQ+ EE difference.

Note in Fig 1A that the FQ^+^ proxy method compared to the standard RQ^+^ equation exhibits more apparent variability and bias than does the plot of the RQ^-^ versus RQ^+^ equations depicted in Fig 1B. Regression does not, in general, properly address agreement (explained in (23)). Fig 2 depicts agreement between the FQ^+^ and RQ^+^ methods (Fig 2A), while agreement between the RQ^-^ and RQ^+^ equations is presented in Fig 2B.

The performance of the RQ^-^ method was strikingly superior to that of the FQ^+^ method; indeed, the ± 2 SD range of agreement was 18.8-fold greater for the FQ^+^ equation than for the RQ^-^ method. This is notable, in part, because the data involve 24h averages for which one expects RQ and FQ to be equivalent in unstressed weight stable animals (as was the case in our study). The increased error reflects the fact that RQ is very labile in fed animals (example below).

The RQ^-^ equation exhibited a very slight positive bias (mean bias = 0.00047 kcal/h, representing just 0.11% of the overall mean of the two methods (and 0.11% of the mean RQ^+^ EE since the overall means were equal); this simply indicates that, as expected, the RQ^-^ method exhibited a very slight tendency to overestimate EE compared to the RQ^+^ method. The upper bound for agreement (mean bias + 2 SD) was 0.00088 kcal/h and equates to an error of just 0.21% of the mean. Indeed, of the entire data vector, just 5 of the 168 (RQ^-^ minus RQ^+^) difference values (3%) exceeded the 0.21% limit. Thus agreement between the RQ^-^ and RQ^+^ equations was highly satisfactory.

As predicted by Eq 6, Fig 2B depicts a slight positive relationship between the EE difference and corresponding mean EE calculation. Mixed model regression quantified this as 0.0017 ± 0.00023 kcal/h per kcal/h (p<0.0001).

Another view of the impact of RQ on the Weir RQ^-^ minus Weir RQ^+^ EE difference is depicted in Fig 2C (note rescaling of y-axis); this illustrates that, as predicted, the mean difference between the RQ^-^ and RQ^+^ methods increases as RQ decreases (see Materials and Methods); however, the practical consequence is trivial as the predicted mean EE difference at RQ = 0.7 is just 0.003 kcal/h, representing <0.02% of the corresponding RQ^+^ EE calculated for RQ = 0.7 using Eq 1.

**Fig 3:**
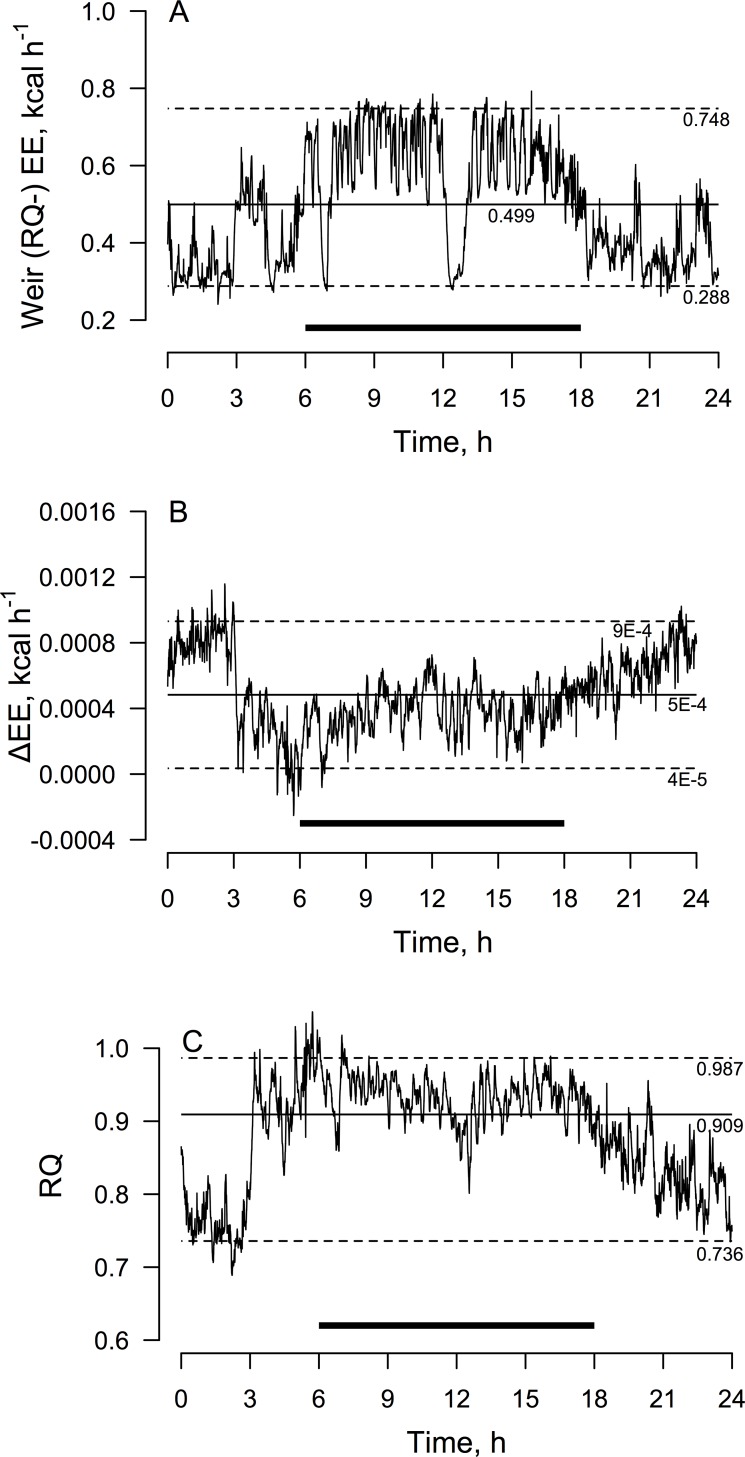
Time plots of continuous EE data calculated using the Weir RQ^-^ method and its correspondence to the standard Weir RQ^+^ method and RQ in a single mouse. The thick solid lines indicate the dark photoperiod. (A) EE as computed by the RQ^-^ equation (see text or Fig 1). Median, 97.5th and 25th percentiles are indicated. (B) The RQ^-^ minus RQ^+^ EE difference. Mean ± 2 SD limits are indicated. (C) RQ. Median, 97.5th and 25th percentiles are indicated. Note that the Δ EE trace indicates remarkably little error between the RQ^-^ and RQ^+^ equations, but that it tends to rise to its most extreme values as RQ decreases. This fits with the predicted behavior of the RQ^-^ equation. EE, energy expenditure; RQ, Respiratory Quotient; Δ = Weir (RQ^-^) minus standard the Weir (RQ^+^) EE difference.

The foregoing data indicate that the Weir RQ^-^ method works very well for analyzing EE data averaged over longer time durations, but we also wanted to confirm that this method holds up for continuous time series data in single mice. The data depicted in Fig 3 demonstrate that it does.

Of particular note in Fig 3A, when EE tended to be lowest during the light photoperiods, the RQ^-^ method tended to deviate furthest from the RQ^+^ method (Fig 3B), and this alignment corresponds to the periods of lowest RQ (Fig 3C). Indeed, the EE difference time series in Fig 3B and the RQ time series in Fig 3C embody symmetrical mirror image-like profiles. This is fully in keeping with the predicted behavior of the RQ^+^ method as explained above.

We also performed the time series analysis using the standard Weir equation assuming the mouse diet’s FQ of 0.91; the difference between the upper and lower agreement limits was more than 30-fold greater than for the RQ^-^ method (0.0275 vs. 0.0009 kcal/h). This emphasizes the fact that the difference between FQ and RQ in ad-lib fed animals varies markedly across the circadian cycle and so reduces the accuracy of EE calculations when using FQ in place of measured RQ.

**Fig 4:**
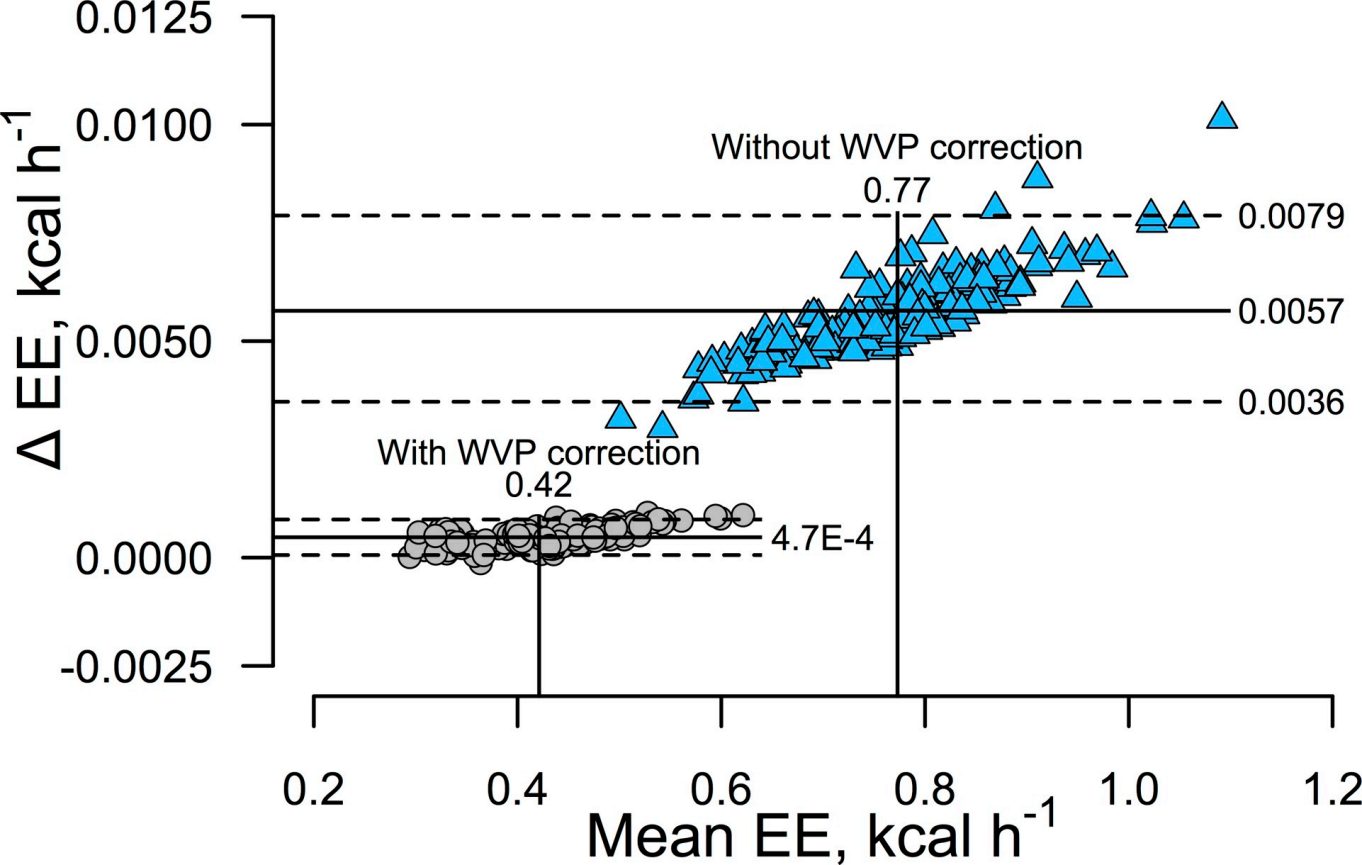
Effect of not correcting for water vapor dilution on mean EE and agreement between the RQ- and RQ+ methods for calculating EE. Data consist of 168 measurements of 24h EE in n = 8 mice.

It is important to emphasize the importance of correcting fractional gas concentrations for WV dilution. Fig 4 demonstrates that failure to correct for WV artifactully increases EE by a large amount. Failure to correct for WVP will also badly distort RQ calculations, and constitutes one reason that RQ can differ from FQ. In the present work, mean 24h RQ calculated after correction for WVP was 0.895 ± 0.0007 and did not differ from the calculated FQ of 0.906 (p = 0.15). Failure to correct for WVP resulted in a mean RQ of 0.43 ± 0.004. We believe that correcting for WVP is ideally done using Dalton’s law of partial pressures because physically or chemically removing WV from airstreams involves considerable potential for error and increases calorimeter response times [3] (discussed below).

### Accuracy of RQ^-^ method depends on the incurrent oxygen fraction

Eq 5 immediately reveals two additional insights: The first is that if F_i_O_2_ equals the coefficient of the RQ term in the numerator of the ratio in Eq 5 (i.e., 0.2191), then that ratio equals exactly 1.0 across the entire range of RQ because the numerator and denominator will be identical linear equations. Therefore, manipulating F_i_O_2_ can, in theory, result in perfect agreement between the RQ^-^ and RQ^+^ equations. The second insight, a corollary of the first, is that if F_i_O_2_ deviates substantially from normoxia, then Eq 5 will agree less well with the standard method. It should be noted in this context that F_i_O_2_ can be generated at a ‘customized’ value by blending gases with precision flow controllers, e.g., as in [18–26].

## Discussion

### Agreement

The present work demonstrates clearly, and to our knowledge for the first time, that the ‘RQ-free’ method published seven decades ago by Weir [6] calculates EE with almost no error compared to Weir’s standard method that uses both VO_2_ and RQ. Indeed, our finding that the maximum observed relative error of the RQ^-^ method was just 0.21% may seem difficult to reconcile with the fact that RQ is universally acknowledged as an important variable for transforming VO_2_ into EE (e.g., [2, 3, 6, 7]). Our data by no means challenge that view, rather the reconciliation, as mathematically demonstrated in the Materials and Methods, is that both VO_2_ and the EE transform that constitute Eq 1 contain functions of RQ that very nearly cancel each other out. This serendipitous property of aerobic biomathematics means that the product of VO_2_ and the transform equation can be simplified into an expression that excludes any explicit dependence on either RQ or VO_2_.

Because the RQ^-^ approach constitutes a method rather than a single equation, we should stress how easy it is to adapt it for use with a different RQ^+^ equation, for instance the equation derived by Hall and associates [7]. Adjusted for a typical contribution of dietary protein to human metabolic rate (15% of EE) the Hall equation is:

$$EE=VO_{2}(3.85+1.07RQ)$$ [8]

Note that Eq 8 indicates that 1 L of O_2_ corresponds to 4.92 kcal when RQ = 1.0; therefore, this is the constant for the RQ^-^ equivalent:

$$EE=4.92\times {\dot{V}}_{e}\times \Delta FO_{2}$$ [9]

When Eqs 5 or 9 are derived to use the incurrent flow rate V_i_ instead of V_e_, the result is simply to replace V_i_ for V_e_.

### Adjusting respirometry for protein metabolism

Protein oxidation has been widely ignored in research involving respirometric EE estimation. Depending on study design and goals, the potential impact of protein metabolism may or may not be significantly problematic. To illustrate, we note that the predicted ‘error’ in EE calculated from the RQ^+^ or RQ^-^ methods *without* adjustment for the high dietary protein percentage of our study diet would be a non-trivial 2.3%. Accordingly, it would seem problematic to ignore protein when comparing EE in groups fed diets that differ markedly in protein content, or in studies involving interventions that might alter protein metabolism.

One reason that protein metabolism has been ignored is the assumed need to measure nitrogen excretion (6.25 g of protein metabolized per g of excreted nitrogen [7]). This requires a specialized metabolic cage to collect urine for nitrogen determination. By contrast, the simple approach of estimating protein metabolism from diet composition has been implemented in excellent work involving the measurement and mathematical modeling of EE [7, 27–29], and we note that protein is taken into account in calculating FQ, which, in turn, is expected to agree with RQ, as it did in the present study. The point is that investigators should consider adjusting respirometric EE calculations for protein metabolism based on diet composition.

### Advantages of the RQ^-^ method

Our findings do more than simply validate the use of the RQ^-^ method for research involving the need to compute only EE. In particular, measuring VCO_2_ is technically problematic and adds cost. CO_2_ calibration gases of <1% accuracy are not widely available and most are accurate to only ±2–5%. CO_2_ is generally measured using an optical absorption method with non-linear properties [3], and CO_2_ analyzers vary widely in the extent to which they successfully compensate for this. Some investigators use multiple span gases to address residual non-linearity, but this introduces further calibration uncertainty because each span gas has an independent and unknown error percentage. Operational issues also arise: a researcher might employ an unreliable or poorly calibrated CO_2_ analyzer, or the tank of CO_2_ span gas may prove to be empty immediately prior to an important experiment.

By contrast, it is considerably easier to accurately measure fractional O_2_ concentrations within a range narrowly centered on the normal atmospheric value. One reason whose importance is hard to overstate is that O_2_ calibration is anchored to the atmosphere’s near-constant FO2 of 20.939 ± 0.0003% after correcting for WVP and variations in BP [10]. Another crucial advantage is that the O_2_ calibration curve is linear. Finally, modern high precision O_2_ sensors are exquisitely sensitive to fractional O_2_ concentrations.

Our results and comments regarding CO_2_ should make it obvious that the RQ^-^ method provides an excellent means by which to rescue high accuracy EE data in studies compromised by faulty CO_2_ sensing. Moreover, the RQ^-^ method may also be developed to provide a way to determine whether CO_2_ sensing is potentially compromised. Specifically, if CO_2_ sensing is accurate, then mean EE as calculated by the RQ^-^ method will be only very slightly higher than EE calculated by the RQ^+^ method (0.11% in the present study), whereas if, for example, CO_2_ sensing is too low and therefore results in artifactually low RQ values, then mean EE as calculated by the RQ^-^ method may be notably higher than EE calculated by the RQ^+^ method. To take advantage of this quality control approach, it would be important to first determine typical RQ^-^ to RQ^+^ EE ratios in studies where the gas sensors are known to be functioning well.

Another potentially important application of the RQ^-^ approach is that eliminating the CO_2_ sensor and related components would help minimize the weight, volume and expense of small ‘wearable’ calorimeters designed to measure human EE during occupational, recreational and ‘everyday living’ tasks.

Finally, our study also lends credence to the use of the Weir RQ^-^ method in previous research involving measurement of EE during exposure to nitrous oxide, a gas that interferes with CO_2_ sensing [18–26].

## Supporting information

S1 Dataset 24h_mean_EE.xlsx. (XLSX)

S2 Dataset Mouse6. (XLSX)
